# A Photolysis-Assist Molecular Communication for Tumor Biosensing

**DOI:** 10.3390/s22072495

**Published:** 2022-03-24

**Authors:** Yue Sun, Huafeng Bian, Yifan Chen

**Affiliations:** 1 School of Mechanical and Electrical Engineering, Chengdu University of Technology, Chengdu 610000, China; sunyuestc90@126.com (Y.S.); 2019050603@stu.cdut.edu.cn (H.B.); 2 School of Life Science and Technology, University of Electronic Science and Technology of China, Chengdu 610000, China; 3 Yangtze Delta Region Institute (Huzhou), University of Electronic Science and Technology of China, Huzhou 313000, China

**Keywords:** biosensing, intersymbol interference, molecular communication, synchronization

## Abstract

Molecular communication (MC) is a promising bioinspired paradigm for exchanging molecule information among nanomachines. In this paper, we propose a synchronization-assist photolysis MC system that aims to transmit the biosensing signal of the tumor microenvironment, facilitated by mitigating redundant molecules for improved bit error rate (BER) performance. Benefits from biocompatible MC, biosensors could transmit biosensing signals of the tumor in vivo instead of converting them to electrical signals. Due to diffusion motion’s slow and stochastic nature, intersymbol interference (ISI), resulting from previous symbols’ residual information molecules, inevitably occurs in diffusion-based MC. ISI is one of the challenges in diffusion-based MC, which significantly impacts signal detection. Inspired by on–off keying (OOK) modulation, the proposed modulation implements a switch of molecules and light alternatively. The light emitted is triggered by a synchronization signal, and the photolysis reactions could reduce the redundant molecules. An expression for the relevant channel impulse response (CIR) is derived from a hybrid channel model of diffusion and photolysis reaction. In this paper, we implement the maximum posterior estimation scheme to find the optimal decision threshold and analysis the BER performance in terms of different time intervals of the system. Numerical simulations demonstrate that the proposed method can improve the channel capacity and BER performance. We believe that our work may pave the way for MC application in biosensing.

## 1. Introduction

Molecular communication (MC) has attracted considerable research attention due to its potential application in areas such as drug delivery systems, biosensing [1], Internet of Bio-Nano Things (IoBNT) [2], and body area networks [3]. Note that the IoBNT, a typical Internet-of-Things (IoT) scenario, emerges as a shift paradigm concept for communication and network engineering, which stems from synthetic biology and merges the interconnected biosensors nanomachines, and biocomputing devices [4]. IoBNT provides novel IoT applications in nanoscale, such as health monitoring, biosensing, tumor detection and targeted therapy. However, the fundamental information exchange technology behind IoBNT is MC. Bioinspired MC is a promising communication paradigm in the body with the advantages of size, power efficiency, and, most importantly, biocompatibility. Whilst there is still a gap between the theoretical and practical applications to human health, it is critical to motivate the continued growth of applications of MC theory [1]. MC holds the considerable potential to enable biosensing and propagates the information in vivo through the biochemistry signal underpinning the human body and cells. Studies over the past two decades have provided significant insights on properties changes in the presence of a tumor and corresponding tumor biosensing [5]; however, up to now, little attention has been paid to transmit this biosensing information in MC. In this paper, we aim to develop a synchronization-assist photolysis MC system that transmits biosensing signals of the tumor microenvironment metastasis (TMEM), such as pH conditions.

Diffusion, molecules in a fluid propagating via Brownian motion, is the basic model for MC. Brownian motion is the stochastic motion of molecules in a fluid medium, which leads to the information transmitted previously arriving at the receiver after the current arrival molecules, causing the intersymbol interference (ISI), limiting the transmission rate of MC. Indeed, in avoiding the ISI, we could send a date over long time intervals and lower the transmission rate. However, the real-time biosensing in TMEM requires a high transmission rate, as detailed discussed in Section 2. Thus, it is fundamental to mitigate the ISI in MC for real-time tumor sensing. There is a growing body of literature that recognizes the importance of mitigating ISI in MC [6,7]. The above methods can be divided into two types, passive strategies, not eliminating the surplus molecules directly instead shaping the channel impulse response (CIR) symbols [8,9], while active strategies, in which the redundant information molecules are degraded by the enzymes [10] or light [7] so that the receiver can not recognize them.

Kuran et al. proposed Concentration Shift Keying (CSK) and Molecule Shift Keying (MoSK) modulation in [8]. The CSK modulation implements the received molecules’ concentration as the amplitude of signal; for example, high concentration represents the signal ‘1’, and low concentration indicates signal ‘0’. In comparison, MoSK modulation uses different types of molecules to represent the signal. The authors argue that compared with CSK, MoSK considerably reduces the ISI since a different molecule type represents each bit. Yan et al. [11] proposed a derivative-based scheme to detect the symbol by the derivative of CIR, which has a much shorter peak time than CIR itself. The tail, therefore, vanishes much faster than that of CIR, resulting in reducing ISI. In [9], Kuran et al. proposed a nonlinear mapping method in MC, which reduces the delay profile of the sampled data to mitigate ISI at the receiver. However, adopting the above passive strategies, the redundant information molecules causing ISI are not eliminated directly, which limits the transmission rate [10]. Meanwhile, the computational complexity required is high, which is a considerable burden on the limited capability of nanomachines. Thus in [10], Noel et al. implemented enzymes to catalyse the degradation of molecules in the medium. Enzymes selectively transform the molecules so that they become unrecognisable by the receiver, which mitigates ISI, but the enzymes presented around the receiver could reduce the strength of the signal. In [7], Dambri et al. introduced photolysis reactions, in which the emitted molecules are transformed by light instantly. The light is required to be emitted at the optimal time. However, because light emissions time is strictly limited at the peak time of each slot, it is necessary to introduce the synchronization mechanism to ensure that the light is emitted at the right time.

Due to the low-speed and stochastic propagation characteristics, precise synchronization in transmitters and receivers is particularly challenging in diffusion-based MC [12]. In response to this issue, some synchronization detection schemes have been proposed and divided into two types using two different types of molecules [13,14] and using signal processing method instead [12,15]. Jamali et al. [13] assumed a synchronization frame that utilizes two types of molecules, type A for information molecules and type B for synchronization molecules, facilitating simultaneous symbol and synchronization detection; however, the exact types of specific molecules are not mentioned in [13]. While in [14], Mukherjee et al. introduced a block synchronization technique by utilizing two types of molecules with different diffusion coefficients for synchronization and information transmission. As different from [13,14], Noel et al. [15] introduced a simple asynchronous detection scheme without requiring multiple molecule types and detectors, which is based on peak concentration detection. Following the idea of [15], Tung et al. [12] derived an approximate maximum-likelihood delay estimator and a decision feedback equalizer to mitigate sensitivity to synchronization errors; however, it requires a more complex scheme in the receiver. It is important to note that synchronization detection occurs at each release time for all the aforementioned works.

In this paper, we propose a synchronization-assist MC for biosensing that hybrids photolysis and molecules diffusion. The information molecules diffuse to the receiver, then degraded by photolysis when reaching around the receiver. The synchronization molecules are employed to trigger the receiver to emit light impulses during symbol “0” transmission. Then the photolysis reaction degrades the redundant information molecules to mitigate ISI. Because the emitting light time starts almost at the beginning of the symbol “0” slot, instead of calculating the peak time of the received signal, it can be adjusted with the change of the symbol period. The synchronization mechanism makes sure that it is easier to achieve precisely. In two scenarios, diffusion without photolysis and diffusion with photolysis, expressions for CIR are derived. Then we present the relationship between the channel capacity and the peak time by simulation and obtain the optimal symbol duration to achieve the maximum channel capacity.

The rest of this paper is organized as follows. In Section 2, we introduce our system model, including tumor biosensing, molecules propagation, photolysis reaction, and diffusion with photolysis. We evaluate the system’s performance and derive the bit error rate (BER) and channel capacity in Section 3. In Section 4, we present and discuss numerical and simulation results. Conclusions and the ongoing direction of our research are described in Section 5.

## 2. System Model

### 2.1. Biosensing for Tumor

In the presence of a tumor, several biological properties changes have been observed in the microenvironment of the peritumoral region, which includes fluctuations in blood velocity [16], changes in oxygen levels [17], temperature variations, reshaping of the fibrosis vasculature [18], and alterations in pH profile [5]. In [19], Jelski et al. report Several brain-tumor-derived biomarkers accumulate in the blood or cerebrospinal fluid, including nucleic acids, proteins, small molecules, such as H${}_{2}$O${}_{2}$ [20], specific amino acids [21], and extracellular vesicles.

Dissemination of tumor cells is a vital step in tumor metastasis [22]. Thus, the real-time dissemination signals directly correlated with metastasis are indisputable evidence for tumor biosensing. Therefore, as reported in [22], using intravital microscopic imaging (IVM), Harney et al. found that transient vascular permeability and tumor cell intravasation occur simultaneously and exclusively in the tumor microenvironment of metastasis (TMEM). Furthermore, with the advancements in bioluminescence imaging and bioluminescent reporters in IVM, Liu et al. [23] demonstrated that it is capable of imaging signalling dynamics in real time in the TMEM. The rapid proliferation of tumor cells causes an insufficient supply of blood, which results in a lack of oxygen and other nutrients. As a result, cancer cells produce lactic acid, which contributes to a lower pH in tumors than in normal tissue (pH = 7.4) [5]. Consequently, these pH-responsive and real-time changes in TMEM require a high transmission rate to deliver the information for biosensing.

Such real-time changes in conditions of the microenvironment could be regarded as time-variant biological gradients induced by specific tumor. It is essential to sense the information towards the biological gradients for tumor detection. Nanoparticles can be designed to respond to pH by changing surface chemistry, changing particle size or shape, disassembling or releasing cargo in [24]. The last few years have seen many breakthroughs concerning finding pH-responsive nanoparticles with extremely low toxicity toward healthy cells [25]. For instance, self-assembled micelles [26], which are stable at a physiological pH of 7.4, swell in acidic environments, such as intracellular (pH = 6.8) and endosomal (pH = 5.5). Moreover, as indicated in the review [24], synthesizing pH-responsive nanoparticles has attracted significant interest in drug delivery systems since nanoparticles could be internalized into cells via acidified vesicles. Meanwhile, the result also demonstrated that pH-responsive polymer polymer-modified nanoparticles could enhance drug loading and release rate of the anticancer drug 5-fluorouracil as reported in [27]. In [28], Barnoy et al. realise the AND, OR, NAND, NOR, XOR, and XNOR gates by the manufactured NP-OG constructs, which conjugates the pH-responsive Oregon Green 488 (OG) to the nanoparticles with a trypsin-cleavable peptide. The constructs allow for efficient fluorescent biosensing with logic gates [28]. In [29], the DNA-grafted hemin was utilized not only as of the module for dynamic DNA assembly but also as the tunable mimetic enzyme; then, activated hemin-mimetic enzymes can provide a simple, fast and signal amplification strategy for fluorescent biosensing. To facility the target-triggered DNA self-assembly catalytic system, the fluorescent spectra can be generated [29]. We follow this work, adopted as a component in the receiver, in which the specific molecules trigger fluorescent generation. The pH conditions detected by pH-responsive NP-OG constructs could be coded as the information to be transmitted.

### 2.2. Provitamin D3 Molecules Propagation Process

In this paper, we assume that MC operates in a medium with an unbounded 1-D fluid environment, representing the tissues surrounding the tumor.

As information molecules, provitamin D3 can be photodecomposed by UV light. However, the nonphotodecomposed synchronization molecules can trigger the fluorescent. The transmitter releases impulses of provitamin D3 molecules, with a number *Q*. We employ binary modulation with constant bit interval, where information molecules are released at the start of symbol “1”, while synchronization molecules are released during the symbol “0”. As different from conventional MC, where “1” or “0” are generated randomly, the symbol “1” or “0” here represents the coded pH information. A trigger signal can be generated once the receiver detects the synchronization molecules with a higher diffusion coefficient. Receivers are light-emitting components activated by trigger signals to emit light impulses of a specific wavelength when the transmitted symbol is “0”. The information molecules released by the transmitter can be photodecomposed by the light impulses; however, the nonphotodecomposed synchronization molecules can trigger the fluorescent.

**Figure 1:**
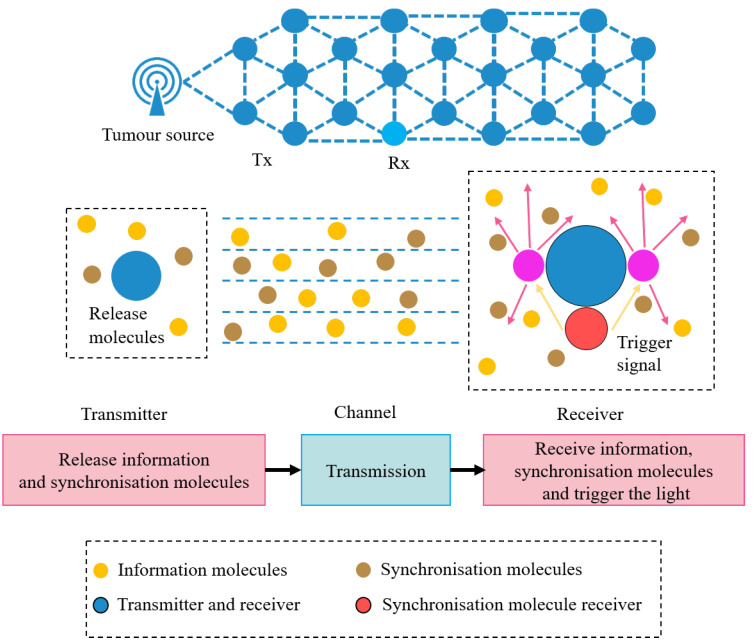
Schematic of physical model, Tx denotes transmitter, and Rx denotes receiver.

The MC system in the IoBNT for biosensing is illustrated in Figure 1. The biosensing signal, generated by the tumor sources, propagates from the transmitter to the receiver. While in the IoBNT network, the nodes are interconnected by way of the MC process, which implements the provitamin D3 molecules as the information carriers. The transmitter releases the information and synchronization molecules; meanwhile, the receiver receives information molecules and then triggers photolysis reactions once detecting the synchronization molecules with a higher diffusion coefficient.

The system is characterized by the assumptions as follows:

(i) the propagation is unbounded,
(ii) the information transmitted is modulated by concentration and UV light,
(iii) the molecule reaching the receiver is considered to be fully absorbed and cannot be recycled.

Molecular diffusion, thermal vibration and collision with other molecules in the fluid environment. The motion of molecules is called Brownian motion, that is, random motion without any priority direction leads to the concentration change of molecules in time and space, which is characterized by Fick’s second diffusion law:

(1) $$\frac{\partial C(d,t)}{\partial t}=D\nabla^{2}C\left(d,t\right),$$

where *D* is the diffusion coefficient, ∇ is the Laplace operator. The diffusion coefficient can be determined by the Einstein relation:

(2) $$D=\frac{k_{B}T}{6\pi \eta R_{p}},$$

where $k_{B}$ is Boltzmann’s constant, *T* is the temperature in kelvin, η is the dynamic viscosity of the fluid, and $R_{p}$ is the radius of the particle.

Because the closed-form analytical solution can not always be attained, we can get the conditional solution by imposing some boundary conditions in the 1-D environment. CIR of the end-to-end channel, denoted by h(t), is defined as the probability of the output molecule at time *t* and at point distance from transmitter *d* when the transmitter is stimulated impulsively at time $t_{0}$ = 0, which can be described as [30]:

(3) $$h_{1}\left(t\right)=\int_{d}^{d+R}\frac{1}{\sqrt{4\pi Dt}}\exp\left(-\frac{x^{2}}{4Dt}\right)dx,$$

where *d* is the distance from the transmitter to receiver, *R* is the receiver radius.

The peak time $t_{p}$ is calculated corresponding to the maximum value of the signal amplitude, which can be obtained by taking the first-order derivative of CIR in pure diffusion. In the 1-D environment, the peak time should be derived from formulation (3), denoted as:

(4) $$t_{p}=\frac{d^{2}}{2D},$$

**Figure 2:**
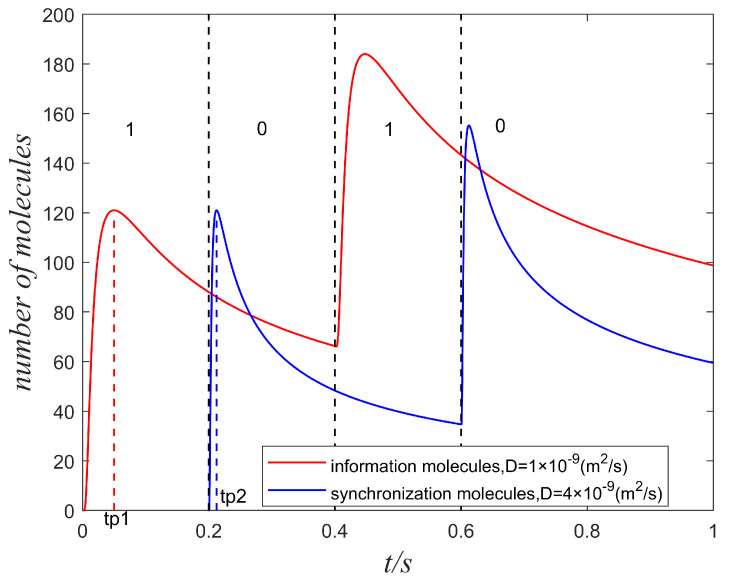
The information molecules and synchronization molecules with different diffusion coefficients are transmitted along with symbol sequences 1010, where information molecules are released at the start of symbol “1”, while the synchronization molecules are released during the symbol “0”. t/s denotes that the unit of time is seconds.

According to Figure 2, the information and synchronization molecules are transmitted at different symbol slots. The high diffusion coefficient can lead to the rapid formation of the peak value so that synchronization molecules can be detected early than information molecules.

### 2.3. Photolysis Reaction

Photodissociation, also known as photolysis, is a reaction in which a photon breaks down a chemical bond in a chemical molecule. This photon’s energy must be high enough for the bond to break beyond its dissociation barrier. Because photon energy is inversely related to wavelength, photons in the infrared spectral region do not have enough energy to produce direct photodissociation of molecules.

The photolysis process is fast and represented by the first-order differential rate equation:

(5) $$\frac{dC}{dt}=-JC,$$

where *C* is the concentration of a particular degradable molecular *X*, and *J* is the photolysis rate coefficient of the molecule. The rate is determined by chemical and environmental factors, such as the photoadsorption properties of the medium, the intensity of the light radiation, the reactive of the target chemical, etc.

An accurate description of these photolysis processes is done by calculating the photolysis rate coefficient, the *J* value, in [31]:

(6) $$J=\int_{\lambda }\sigma \left(\lambda \right)\varphi \left(\lambda \right)F\left(\lambda \right)d\lambda ,$$

For each photoactive molecule with an absorption cross-section σ(λ) and a photolysis quantum yield φ(λ), the *J* value is given by integrating the product over wavelengths λ. Here *F* is the actinic flux.

The photolysis reaction that causes human skin to produce vitamin D daily following exposure to sunshine is the one that has received the most attention. During exposure to sunlight, provitamin D3 is converted to previtamin D3, which is rapidly thermally isomerized inside the plasma membrane to cholecalciferol [32]. Photolysis reaction is one of the most well-known nonenzymatic reactions in our bodies. According to [32], The transition in 7-DHC exhibits a broad absorption line shape spanning the wavelength range of 260 and 290 nm, the optimal irradiation range is in the absorption band of 280–295 nm. That energy breaks a covalent bond, turning provitamin D3 to previtamin D3, which will thermodynamically convert to Vitamin D3 without the need for enzymes. The sensitive molecules to the specified wavelength as information carriers can be chosen from naturally occurring molecules such as chromophores and provitamin D3 or bioengineered ones. The chromophores are visible light-sensitive, while the provitamin D3 is UV-sensitive. However, molecules with a specified wavelength absorption can be engineered, as in the study [33].

It is not harmful to use visible light inside the human body for medical purposes [7]. If shorter wavelengths are employed, specific parameters must be followed to ensure the application’s safety. UV light is safe, but the amount of radiation energy and the length of exposure should be considered to ensure it is not hazardous. Therefore, the American National Standards Institute (ANSI) defined the standard ANSI Z-136.1 [34], which gives the maximum permissible exposure limits (MPEs) for users. The minimum MPE for the UV light is 3.0 mJ/cm${}^{2}$ over 8 h. Therefore, if the energy and the exposure time are safe for the cornea, they should be safe inside the body as well. While the thermal process is the main cause of UV radiation damage [35], to design a safe UV generator is necessary to generate nonthermal radiation by using a pulsed beam instead of a continuous one. The energy will be released in a short time, and this compression delivers the light beam more rapidly, which gives more power using less energy [36]. The shorter the pulse, the less energy is consumed, which greatly reduces the risk of tissue burn. Thus, to safely generate UV light inside the body, the generator should use ps pulses or less, with energy less than 3 mJ/cm${}^{2}$. That can justify the chosen value of the pulse duration, which is 4 ps pulse and 2 mJ/cm${}^{2}$ as incident energy [37].

(7) $$\varphi \left(t\right)=\frac{\gamma }{\sqrt{\pi }T}e^{{\left(-\frac{t}{T}\right)}^{2}},$$

where γ is the number of incident photons per cm${}^{2}$, and *T* is the pulse duration, which are set for the experimental studies.

### 2.4. Diffusion with Photolysis

Enzymes with the advantage of high selectivity for their substrates are catalytic biomolecules. In MC, some enzymes are commonly used to reduce redundant information molecules, ISI, in nature in [38]. For example, acetylcholinesterase is an enzyme in the neuromuscular junction that hydrolyzes acetylcholine as it diffuses to its destination in [39]. Similar to enzymes, the lights of a specific wavelength can photolyze the molecules.

Considering the limitation of light transmission in the body thus, we assume that the photolysis reaction occurs locally around the receiver. In the scenario of photodissociation along with diffusion, the molecular concentration can be expressed as:

(8) $$\frac{\partial C}{\partial t}=D\nabla^{2}C-JC$$

A lower bound is derived on the expected point concentration of *Q* molecules at a distance *d* from the transmitter. The lower bound is

(9) $$C\left(d,t\right)\geq \frac{Q}{\sqrt{4\pi Dt}}\exp\left(-Jt-\frac{d^{2}}{4Dt}\right),$$

We also assume that is satisfied with equality, and the impulse respond is

(10) $$h_{2}\left(t\right)=\int_{d}^{d+R}\frac{Q}{\sqrt{4\pi Dt}}\exp\left(-Jt-\frac{d^{2}}{4Dt}\right)dx,$$

where *d* is the distance from the transmitter, *R* is the receiver radius.

## 3. Receiver Model

In general, the symbol sequence is denoted by $a_{i}\in \left\{0,1\right\},\left\{i=0,1,2,\ldots \right\}$. During the same interval $T_{b}$, the impulse with the number of molecules *Q* are emitted into the diffusion channel for the symbol “1”, denoted $\delta \left(t\right)$, while symbol “0” is denoted by emitting a light impulse. The transmitted signal s(t) at the transmitter is

(11) $$s\left(t\right)=Q\sum_{i=0}^{\infty }a_{i}\delta \left(t-iT_{b}\right),$$

Subsequently, the received signal at the receiver is

(12) $$y\left(t\right)=s\left(t\right)*h\left(t\right)=\sum_{i=0}^{n}a_{i}h\left(t-iT_{b}\right),$$

When noise is taken into account, the received signal can be expressed as

(13) z(t)=y(t)+n(t),

and n(t) denotes the additive white Gaussian noise, which follows a normal distribution.

When *n* symbols “1” are transmitted, then received signal can be expressed as

(14) $$y\left(t\right)=\sum_{i=0}^{n}a_{i}h\left(t-iT_{b}\right)\;\left(a_{i}=1\right),$$

Thus, the current signal observed by receiver in pure diffusion scenario is denoted as:

(15) $$y\left(t\right)=\frac{QR}{\sqrt{4\pi Dt}}\exp\left(-\frac{d^{2}}{4Dt}\right),$$

The concentration throughout the receiver radius is assumed to be uniform and equal [40].

The photolysis reaction always happens after the symbols “1” are transmitted, thus, the signal observed by receiver in photolysis scenario when the previous symbols are “1” of *n* is denoted as:

(16) $$y\left(t\right)=nQ(\sum_{i=0}^{n}\frac{R}{\sqrt{4\pi D(t-iT})}\cdot \exp\left(-\frac{d^{2}}{4D(t-iT)}-J\left(t-nT\right)\right)),$$

The mean expectation and standard deviation for symbol “0” and symbol “1” in the ith symbol interval are represented by $\mu_{0}$, and $\sigma_{0}$, $\mu_{1}$ and $\sigma_{1}$, respectively.

For symbol “0”, the mean expectation is

(17) $$\mu_{0}=\mu_{ISI0}+\mu_{n},$$

and the variance is

(18) $$\sigma_{0}^{2}=\frac{\mu_{ISI0}+\mu_{n}}{R},$$

For symbol “1”, the mean expectation is

(19) $$\mu_{1}=\mu_{s}+\mu_{ISI1}+\mu_{n},$$

and the variance is

(20) $$\sigma_{1}^{2}=\frac{\mu_{s}+\mu_{ISI1}+\mu_{n}}{R},$$

The optimal detection threshold can be calculated by the maximum posterior criterion, the expression is (21). When *z* is above the threshold value, the received signal is judged as “1”; otherwise, the received signal is judged as “0”.

(21) $$z\begin{array}{cc} \; & 1\\ \; & >\\ \; & <\\ \; & 0 \end{array}\frac{\left(2\sigma_{1}^{2}\cdot \mu_{0}+2\sigma_{0}^{2}\cdot \mu_{1}\right)+\sqrt{{\left(2\sigma_{1}^{2}\cdot \mu_{0}+2\sigma_{0}^{2}\cdot \mu_{1}\right)}^{2}-4\left(\sigma_{1}^{2}\cdot \sigma_{0}^{2}\right)\cdot \left(\sigma_{1}^{2}\cdot \mu_{0}^{2}-\sigma_{0}^{2}\cdot \mu_{1}^{2}-2\sigma_{1}^{2}\cdot \sigma_{0}^{2}\cdot \ln\left(\frac{\left(1-p\right)\cdot \sigma_{1}}{p\cdot \sigma_{0}}\right)\right.}}{2\left(\sigma_{1}^{2}-\sigma_{0}^{2}\right)}=\theta ,$$

When symbol “1” sent is misjudged as “0”, the conditional probability function is

(22) $$P\left(0/1\right)=\int_{-\infty }^{\theta }\frac{1}{\sqrt{2\pi }\sigma_{1}}\exp\left[-\frac{{\left(x-\mu_{1}\right)}^{2}}{2\sigma_{1}^{2}}\right]dx,$$

When symbol “0” sent is misjudged as “1”, the conditional probability function is

(23) $$P\left(1/0\right)=\int_{\theta }^{+\infty }\frac{1}{\sqrt{2\pi }\sigma_{0}}\exp\left[-\frac{{\left(x-\mu_{0}\right)}^{2}}{2\sigma_{0}^{2}}\right]dx,$$

The error probability, denoted by $P_{e}$, is expressed as:

(24) $$\begin{array}{cc} P_{e}\; & =pP(0/1)+(1-p)P(1/0)\\ \; & =pQ\left(\frac{\mu_{1}-\theta }{\sigma_{1}}\right)+\left(1-p\right)Q\left(\frac{\theta -\mu_{0}}{\sigma_{0}}\right), \end{array}$$

Molecules diffused from the transmitter to the receiver meanwhile molecules degrade over time. The probability that a molecule transmitted in slot i∈{1,2,…,n} arrives in slot *n* can be expressed as

(25) $$q_{m}=\int_{m\tau }^{(m+1)\tau }f\left(t\right)dt,$$

where m=n−i, f(t) is the probability density function (pdf) of first-arrival time of the molecule [41], in the one dimensional domain assumed in this paper, f(t) is expressed

(26) $$f\left(t\right)=\frac{d}{\sqrt{4\pi Dt^{3}}}\exp\left(-\frac{d^{2}}{4Dt}\right),$$

We thus describe the probabilities of successful bit transmission as follows:

(27) $$P\left[Y_{n}=0|X_{n}=0\right]=P_{n},$$

(28) $$P\left[Y_{n}=1|X_{n}=1\right]=Q_{n},$$

respectively, the false probability is 1−Pn and 1−Qn. Equations (29) and (30) are derived in [42], for n=1, $Q_{1}=q_{0}$, and $P_{1}=1$, that is, communicating a ‘0’ is always successful. For n>2, considering a recurrence relation for Qn and Pn, as follows:

(29) $$Q_{n}=1-\left(1-q_{0}\right)\prod_{i=1}^{n-1}\left(1-pq_{i}\right),$$

(30) $$P_{n}=\prod_{i=1}^{n-1}\left(1-pq_{i}\right),$$

For discrete system, Shannon has figured out the entropy in the case of two possibilities with probabilities ξ and 1−ξ, namely,

(31) $$H=-\xi \log_{2}\xi -\left(1-\xi \right)\log_{2}\left(1-\xi \right),$$

the mutual information in time slot *n* is represented as

(32) $$\begin{array}{cc} I\left(X_{n};Y_{n}\right)\; & =H\left(Y_{n}\right)-H\left(Y_{n}|X_{n}\right)\\ \; & =\chi \left(\left(1-p\right)P_{n}+p\left(1-Q_{n}\right)\right)\\ \; & -p\chi \left(Q_{n}\right)+\left(1-p\right)\chi \left(P_{n}\right), \end{array}$$

where $p=P[X_{n}=1]$ and χ(ξ)=−ξlog2ξ−(1−ξ)log2(1−ξ) are assumed.

The mutual information I(X,Y) achieved in slot n is represented as

(33) $$I\left(X_{n};Y_{n}\right)=H\left(Y_{n}\right)-H\left(Y_{n}|X_{n}\right),$$

The maximum mutual information obtained from slot 1 to *n* is then expressed as

(34) $$C_{n}=\max_{p}\sum_{i=1}^{n}\frac{I\left(X_{i};Y_{i}\right)}{n}\left(\;\mathrm{bits}\;/\;\mathrm{slot}\;\right),$$

## 4. Numerical and Simulation Results

The performance of the proposed scheme is verified by simulations in this section. Parameters in our model are given in Table 1.

First, we evaluated the effect of the photolysis reaction on the ISI.

**Figure 3:**
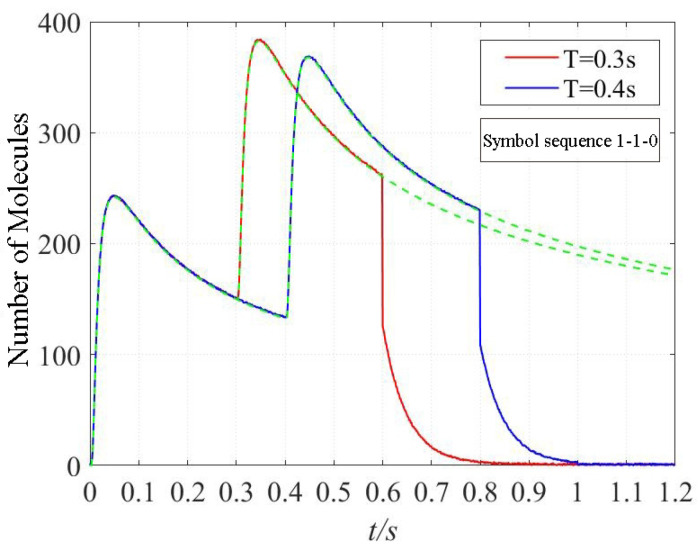
The comparison of CIR curves between the diffusion with photolysis and without photolysis. The solid line denotes the signal when the photolysis reaction happens, while the dotted line denotes the signal when the molecules diffuse without photolysis reaction. t/s denotes that the unit of time is second. The red line denotes a symbol sequence with a time duration of 0.3 s, and the blue line denotes a symbol sequence with a time duration of 0.4 s.

Figure 3 shows the signal amplification decreases rapidly when the photolysis reaction happens because the photolysis reaction accelerates the information molecules degradation, the number of the information molecules observed by the receiver decreases instantly.

**Figure 4:**
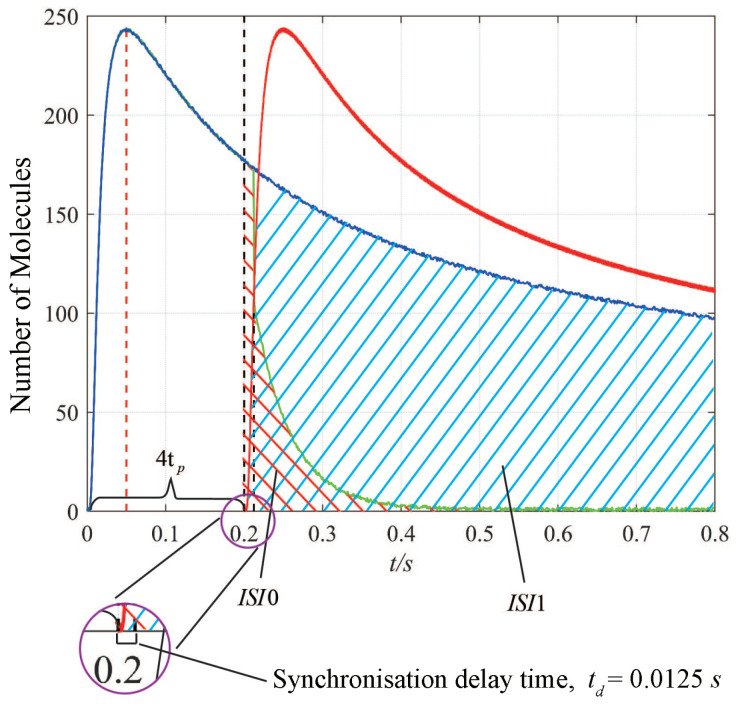
ISI illustrations in two different scenarios, in which the two connected symbols is “11” and “10”. The blue line and the red line denote the received signals when the previous symbol “1” and the current symbol “1”, while the green line denotes the received signal when the previous symbol “1” and the current symbol “0”. The ISI-0 (green shadowed area) denotes the ISI when transmitted symbols are “10”, and ISI-1 (blue shadowed area) denotes the ISI when transmitted symbols are “11”. The time interval of symbols $T_{b}=4t_{p}=0.2$ s.The red dashed line denotes the time to reach the peak concentration $t_{p}=0.05$ s, the black dashed line indicates the synchronization delay time at the current symbol, the delay is $t_{d}=0.0125$ s.

As shown in Figure 4, when the first symbol is transmitted, the amount of ISI depends on the following symbol. During the symbol “0” transmitted, the received signal declines sharply as shown by the green line, which indicates the ISI is mitigated rapidly by the light impulses. Although synchronization molecules with a lower diffusion coefficient can be detected quickly, the delay time is still evaluated. To achieve the large channel capacity and guarantee enough time to receive the information molecules, we set the time intervals between connected symbols $T_{b}=4*t_{p}$.

**Figure 5:**
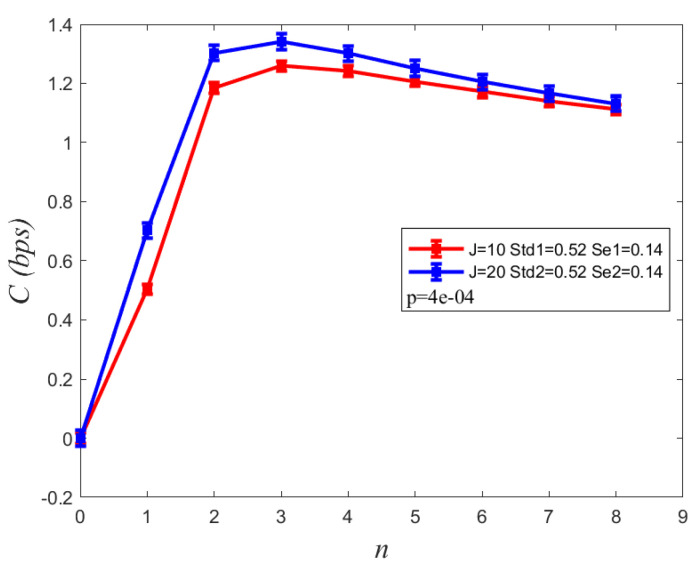
Comparison channel capacity C with the symbol duration $T_{b}$ between two symbols, $T_{b}=n*t_{p}$, *n* = 1, …, 8. *J* denotes the photolysis reaction coefficient. Std and Se denote standard deviations and standard error, respectively.

Figure 5 shows the channel capacity varies with the time duration $T_{b}$ between two symbols along in the different photolysis rate coefficient *J*. We can observe that channel capacity results in a better performance with a higher photolysis rate coefficient. As the time duration T increases, channel capacity increases rapidly and reaches the maximum, when the time duration $T_{b}=3t_{p}$, then decreases gradually. As seen in Figure 5, the standard deviations equals 0.52 and stand error is 0.14. Moreover, as the photolysis reaction is completed rapidly, the changes in channel capacity as a result of increasing the photolysis rate coefficient are not significant. Thereby, it is necessary to demonstrate that this difference is significant using a *p*-value. To further explain the difference of channel capacity between J = 10 and J = 20, we calculated the *p*-value, $4\times 10^{-4}$, which indicates that their difference is significant. To obtain the optimal channel capacity, we can set the time duration $T_{b}=3t_{p}$.

**Figure 6:**
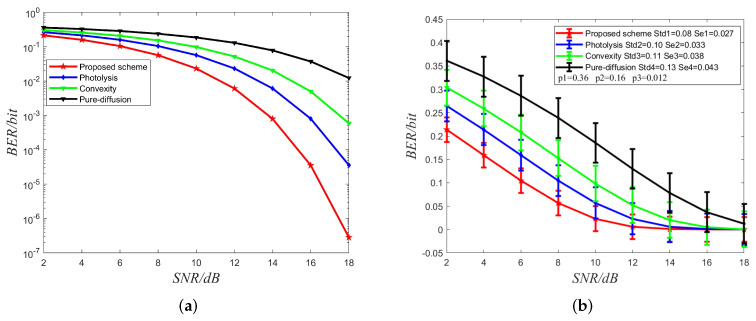
(**a**) Bit Error Ratio (BER) performance comparison of the proposed method with the methods in the previous reference. dB denotes the unit of signal-to-noise ratio (SNR). (**b**) Std and Se denote standard deviations and standard error, respectively. p1, p2 and p3 denote the *p*-values of the comparison between the proposed method with photolysis, convexity and pure diffusion, respectively.

In Figure 6, the BER performances vary with SNR for four methods mitigating ISI are plotted. The red line denotes the proposed method in this paper that adopts photolysis reactions to mitigate redundant molecules with synchronization precision. In contrast, the blue line indicates that the corresponding method adopts photolysis reactions without synchronization. The green line represents the method proposed in [43], which indicates the signal’s convexity. The black line indicates the pure diffusion method. We obtain the simulation results in the same parameters. For fairness, we selected the more significant D as the diffusion coefficient of information molecules to compare the different strategies. Above all the methods, we observe that the BER decreases when the SNR increases. Since the proposed method mitigates the redundant molecules at the receiver side and also benefits from synchronization precision, the results indicate that the method proposed has better BER performance than the rest methods. The *p*-value of the proposed method with other methods also indicates that the proposed method is significantly effective in reducing BER when the SNR is the same. Meanwhile, The detection threshold could be decreased along with the ISI becoming small, which is helpful to improve the system performance. The simulation results validate the efficiency of the method proposed in the paper.

**Figure 7:**
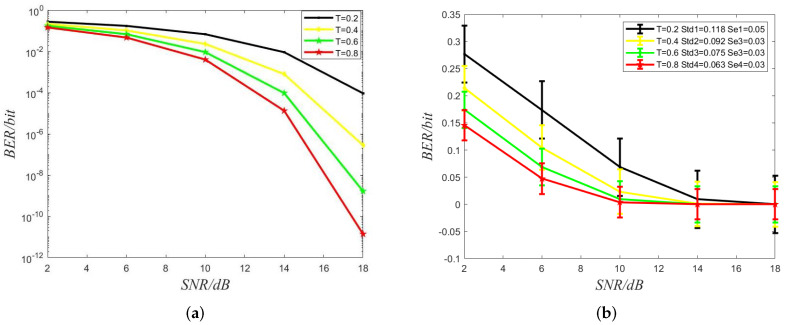
(**a**) Bit Error ratio (BER) versus signal-to-noise ratio (SNR) with the different symbol interval. dB denotes the unit of SNR. (**b**) Std and Se denote standard deviations and standard error, respectively.

Figure 7, four lines have been plotted, which denote four different symbol duration, respectively. We observe that the BER decreases as SNR increases, and the BER decreases when the symbol interval increase. More molecules from previous symbol slots have more time to diffuse when the symbol interval increases, the interference to the current symbol, ISI, is less. However, the bandwidth, as the reciprocal of time, decreases, and the channel capacity decreases as the time duration $T_{b}$ increases because the channel capacity is inversely proportional to symbol duration.

**Figure 8:**
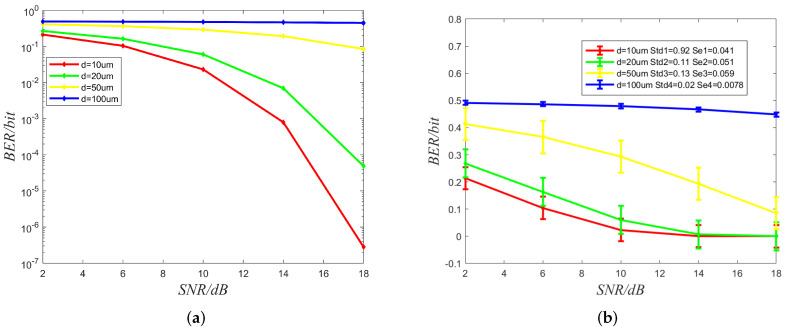
(**a**) Bit Error Ratio (BER) versus signal-to-noise ratio (SNR) with different distances. dB denotes the unit of SNR. (**b**) Std and Se denote standard deviations and standard error, respectively.

Figure 8, the curves are the BER performance with the different distances between transmitter and receiver. It is observed that the BER performance improves as SNR increases; meanwhile, the performance of BER declines significantly as transmission distance increases. Due to diffusion’s slow and stochastic nature, the signal amplification decays heavily as the distance between transmitter and receiver increases. Thus, It indicates that multiple transmitter–receiver pairs, as relays, will be used to achieve long-distance signal transmission as to the IoBNT network.

## 5. Conclusions

In this paper, we propose a synchronization-assist MC for tumor biosensing by mitigating the ISI for improved BER and channel capacity. The information molecules are released when the symbol “1” is transmitted. When the symbol “0” is transmitted, the receiver emits light to degrade the redundant molecules from the previous symbols to reduce the ISI. Because the photolysis reaction happens when the symbol “0” is transmitted, and the symbol “1” is already received, the signal strength of symbol “1” will not reduce. We obtain the optimal symbol duration to achieve the maximum channel capacity. We validate the improved performance of the system by simulations. Future work will consider the biological information molecules dynamic in complex vessels and investigate the optimal symbol duration by utilizing the convex optimization to the channel capacity and BER.

## Figures and Tables

**Table 1:** System parameters used for simulation.

Parameters	Symbol	Value
Released molecules	Q	10,000
Information molecules diffusion coefficient (m${}^{2}$/s)	D	$1\times 10^{-9}$
Synchronization molecules diffusion coefficient (m${}^{2}$/s)	D	$4\times 10^{-9}$
Distance (μm)	d	10
Degradation rate (s${}^{-1}$)	J	20
Symbol interval (s)	Tb	0.2
Signal-to-noise ratio(dB)	SNR	2–18

## Data Availability

The experimental data is contained within the article.
